# Prevalence and Factors Associated With Sexually Transmitted Infections Among Women of Reproductive Age in Tanzania: A Cross‐Sectional Analysis of National Data

**DOI:** 10.1002/hsr2.70807

**Published:** 2025-04-29

**Authors:** Fabiola V. Moshi, Jovin R. Tibenderana, Thadei Liganga, Jomo Gimonge, Sanun Ally Kessy

**Affiliations:** ^1^ Department of Clinical Nursing, School of Nursing and Public Health University of Dodoma Dodoma Tanzania; ^2^ Department of Public Health St. Francis University College of Health and Allied Sciences Ifakara Morogoro Tanzania; ^3^ Department of Otorhinolaryngology Kilimanjaro Christian Medical Centre Moshi Tanzania; ^4^ Directorate of Research and Training Benjamin Mkapa Hospital Dodoma Tanzania; ^5^ Ifakara Health Institute Dar es Salaam Tanzania

**Keywords:** factors and Tanzania, sexually transmitted infections, women of reproductive age

## Abstract

**Background and Aims:**

Sexually Transmitted Infections (STIs) remain a significant public health concern, particularly among women of reproductive age (WRA) who face heightened vulnerability. Despite remarkable advancements in medicine, 1 million new cases of STIs are recorded daily. In Tanzania, however, data on STIs remains limited. This study seeks to bridge that gap by determining prevalence and factors associated with STIs among WRA in Tanzania.

**Methods:**

This was analytical cross‐sectional study among WRA in Tanzania, using data from the current Demographic and Health Surveys (DHS) 2022. 15,254 weighed sample was analyzed using STATA version 18. Analysis considered the complex survey design through application of weights, clustering and strata. Modified Poisson regression models estimated the factors associated with STIs among WRA in Tanzania. Results were presented using adjusted relative risk (ARR) with a 95% confidence interval.

**Results:**

The prevalence of STIs among WRA in Tanzania was 4.7%**.** After adjusting for other variables, the following factors remained significantly associated with STIs among WRA: women aged 25–34 (ARR = 1.55, 95% CI:1.22–1.95), women from rich household (ARR = 1.39, 95% CI: 1.02–1.89), marriage (ARR = 0.81, 95% CI:0.65–0.99) and multiple sexual partners (ARR = 1.80, 95% CI:1.24–2.63).

**Conclusion:**

Although STIs have a relatively low prevalence (4.7%) among Tanzanian WRA, behavioral change campaigns, young adult‐friendly health services, routine screening and tailored preventive education are crucial to achieving universal health goals, reducing new infections, and fostering the overall well‐being of this vulnerable group.

## 1 Background

Despite advancement in medicine and awareness campaigns, the global burden of sexually transmitted infections (STIs) continues to be of concern with over 1 million STIs acquired every single day, many of them being asymptomatic. Annually, an estimated 374 million new infections occur globally, with a quarter of them being curable through timely and appropriate treatment. Among individuals aged 15–49 years, approximately 500 million are estimated to have genital infections with herpes simplex virus (HSV) worldwide [1, 2]. The most common reported and curable STIs include chlamydia, gonorrhea, syphilis and trichomoniasis [1].

In comparison to other regions, Sub‐Saharan Africa (SSA) shoulders a disproportionate load of STIs, accounting for approximately 40% of the global burden with an estimated 60 million new cases of gonorrhea, chlamydia, and trichomoniasis emerging each year [3, 4], and the highest prevalence and incidence rates exhibited in gonorrhea and syphilis [5]. In Tanzania, approximately 1.7 million people are living with HIV (PLHIV), while over 2 million individuals are infected with viral hepatitis B and C combined [6].

STIs pose significant health risks, potentially leading to a range of consequences such as pregnancy complications, pelvic inflammatory disease, cervical cancer, and infertility [3]. Furthermore, evidence indicates that STIs not only increase the risk of acquiring and transmitting HIV but also contribute to increased morbidity and mortality rates [7]. STIs also exert a substantial economic burden, contributing to at least 17% of economic losses resulting from illness. This economic impact extends further with additional costs incurred for the treatment of STIs [8].

The recommended approach for diagnosing and managing STIs at the initial point of contact in healthcare settings in Tanzania is syndromic case management (SCM), which relies on identifying specific symptoms and clinical signs associated with STIs for diagnosis and treatment [9]. Various factors have been reported to be associated with STIs namely, age, multiple sexual partners, condom use, alcohol or drug use, wealth index, marital status, residence, media exposure and education level [2, 3, 10, 11, 12, 13].

Women, particularly those of child bearing age face a heightened susceptibility to STIs than men due to various factors. Gender disparities, including limited access to education and economic opportunities, may lead to risky sexual behaviors such as transactional sex and reduced ability to negotiate condom use during sexual encounters [14].

Effective public health efforts targeting the prevention, control, and appropriate management of STIs necessitate precise evidence regarding the associated factors. Research on STIs in Tanzania is limited, previous researchers based on the prevalence of STIs [2, 8, 15, 16, 17, 18], little is known about factors associated STIs in Tanzania. This foundational knowledge plays a crucial role in shaping educational programs that improve understanding and emphasize evidence‐based, lifestyle‐focused interventions for STI transmission, while also encouraging early detection and treatment. It's crucial therefore to determine prevalence and factors associated with STIs among WRA in Tanzania.

## 2 Methodology

### 2.1 Study Period and Setting

The research utilized data from the Tanzania Demographic and Health Survey (TDHS) of 2022, a comprehensive survey conducted every 5 years [19]. Tanzania, the largest country in East Africa, spans 940,000 km^2^, including 60,000 km^2^ of inland water. Located south of the equator, it shares borders with eight countries: Kenya and Uganda to the north; Rwanda, Burundi, the Democratic Republic of Congo, and Zambia to the west; and Malawi and Mozambique to the south. As of 2022, Tanzania, the largest country in East Africa, boasted an estimated population of 61,741,120 [20]. Tanzania has been divided into nine geographic zones, which are not formal administrative units but serve as a framework adopted by the Ministry of Health's Reproductive and Child Health Section. This zonal grouping combines regions to create larger statistical denominators, improving data reliability by reducing sampling errors in zonal‐level indicators.

### 2.2 Study Design and Data Source

This was an analytical cross‐sectional study conducted using nationally representative secondary data. This is the most recent and publicly accessible source of data with a broad coverage of questions on women's data on STIs. TDHS is supported by the US Agency for International Development (USAID), the initiative's execution is overseen by the Ministry of Health (MoH) in Tanzania Mainland and Zanzibar, along with the National Bureau of Statistics (NBS) and the Office of the Chief Government Statistician (OCGS). Technical assistance is provided by ICF International [20, 21].

### 2.3 Data Collection Procedure

The survey's data collection method entails the use of a standardized questionnaire, consistent across all countries, to gather information from women aged 15–49. These questionnaires are translated into the primary local languages of the participating nations. Before deployment, both the translated questionnaires and the original English version undergo pretesting to ensure accuracy. During this phase, field workers engage in interactive discussions of the questions, offering suggestions for enhancements across all versions. Following the pretest phase, a debriefing session is held with the pretest field personnel, and adjustments are made to the questionnaires based on the insights gained from this process [22].

### 2.4 Sampling Technique

The survey employed multistage cluster sampling to gather information on various aspects of population health, including neonatal mortality, health behaviors, nutritional status, family planning, and demographics. Initially, 629 clusters were identified, from which households were selected. Within each cluster, 26 households were systematically chosen to provide representation, resulting in a total of 16,354 households included in the survey. Eligibility for participation was determined based on the presence of all women aged 15‐49 years in the sampled household the night before the interview. Further details regarding the sampling procedure and design have been previously documented [19].

### 2.5 Study Variables

The outcome variable of this study was sexually transmitted infection which was dichotomized as Yes and No, of which participants were asked if they had any STI in the last 12 months. The independent variables were age of the woman, marital status, education, employment, residence (categorized as urban or rural, based on the DHS classification of living areas), wealth index, condom use for the last sex, media exposure and number of sexual partners and history of STI (Measured using self‐reported responses to the question, *“Have you had any STI in the last 12 months?”* (Yes/No)). Heard of STI (Assessed by asking respondents whether they had ever heard of sexually transmitted infections (Yes/No)). Wealth index was re‐categorized from five to three categories combining poorest and poorer as ‘poor’ middle wealth as ‘middle’ and richer and richest as ‘rich’ aligning with previous research practices. Education was categorized into four categories: no education, primary education, secondary education and higher education. A media exposure variable was generated from household which has either television or radio. Additionally, the age of survey respondents was grouped into three categories 15–24, 25–34 and 35–49 years old. Furthermore, mothers' employment status was re‐categorized into two categories ‘working’ and ‘not working’ as previously categorized. The generation of variables, coding and re‐categorization based on previous literature [2, 3, 10, 11, 12, 13].

### 2.6 Data Management and Analysis

Data cleaning and analysis were conducted using STATA version 18.0 (Stata Corporation, College Station, TX, USA). Categorical variables were summarized using frequencies and percentages and numerical variables were summarized using median and interquartile range. The Pearson *χ* ^2^ test investigated the relationship between outcome variable (sexually transmitted infection) and independent variables. The sample was weighted (v005/1,000,000) to address any over‐ and under‐sampling issues, and the survey set(svy)command in Stata was used in the analysis to account for the survey's complex design.

Self‐reported STIs among women of reproductive age (WRA) were assessed based on their response to whether they had experienced an STI in the past 12 months, with percentages analyzed across different geographic zones in Tanzania.

Based on the Akaike Information Criterion (AIC) and Bayesian Information Criterion (BIC), modified Poisson regression models were used to determine the factors associated sexually transmitted infection, as an alternative to logistic regression. The model estimated the relative risk (RR) and 95% confidence interval (CI) for improved precision of parameter estimates, the variables associated at crude regression were entered into adjusted analysis right after stepwise regression to identify independent variables associated with sexually transmitted infection to identify key factors while controlling for potential confounding effects. Statistical significance was indicated at a *p*‐value of 0.05 throughout. The variables reported were those found to be significantly associated based on Adjusted Risk Ratio (ARR).

### 2.7 Ethical Consideration

A formal written request was made to the DHS program, and approval was granted to access and utilize data from http://www.dhsprogram.com. The questionnaire used in the standard DHS was reviewed and approved by the Medical Research Council of Tanzania, the Zanzibar Health Research Institute, and ICF's Internal Review Board (IRB). Before participating in the survey, participants provided either written or verbal informed consent. There was no coercion involved, and stringent measures were implemented to protect all data, ensuring the confidentiality of personally identifiable information [23]. Additional information regarding ethical considerations can be found elsewhere [24].

## 3 Results

### 3.1 Characteristics of Reproductive Age Women by Self‐Reported Sexual Transmitted Infections

Median age of 15,254 women (interquartile range, IQR) was 28 (21–37) years, STIs were prevalent (6.2%%) among women aged 24–34, those residing from urban (5.3%), women with primary education (5.1%), those who were from middle and rich households (5.1%), Among married women (4.9%), WRA who were working (5.2%). Additionally, the proportion of STI was high among WRA who had more than one sexual partner (11.5%) and those were not exposed to media (4.7%) (Table 1).

**Table 1:** Characteristics of reproductive age women by self‐reported sexual transmitted infections in Tanzania (*n* = 15,254) (Weighted).

Study variables	Total, *n* (%)	Sexually transmitted infection, *n* (%)		*p* value
		No	Yes	
*N*	15,254 (100.0)	14,544 (95.3)	710 (4.7)	
Age categories (Years)				
Median (IQR)	28 (21–37)			
15–24	5810	5653 (97.3)	157 (2.7)	< 0.001
25–34	4609	4322 (93.8)	287 (6.2)	
35–49	4835	4569 (94.5)	266 (5.5)	
Residence				
Urban	5446	5159 (94.7)	287 (5.3)	0.090
Rural	9808	9385 (95.7)	423 (4.3)	
Highest educational level				
No education	2450	2346 (95.8)	104 (4.2)	0.214
Primary	8123	7713 (94.9)	410 (5.1)	
Secondary	4467	4281 (95.8)	187 (4.2)	
Higher	213	204 (95.8)	9 (4.2)	
Wealth index				
Poor	5044	4858 (96.3)	185 (3.7)	0.008
Middle	2880	2733 (94.9)	147 (5.1)	
Rich	7330	6953 (94.9)	377 (5.1)	
Marital status				
Unmarried	8624	8238 (95.5)	386 (4.5)	0.275
Married	6630	6306 (95.1)	324 (4.9)	
Employment status				
Not working	5452	5250 (96.3)	201 (3.7)	0.001
Working	9802	9294 (94.8)	508 (5.2)	
Condom used for the last sex (*n* = 12,011)				
No	11,287	10,661 (94.5)	626 (5.5)	0.067
Yes	724	669 (92.4)	55 (7.6)	
Heard of sexually transmitted infection				
No	3336	3336 (100.0)	0 (0.0)	
Yes	11,918	11,208 (94.1)	710 (5.9)	< 0.001
Number of sexual partners				
No	12,108	11,614 (95.9)	494 (4.1)	< 0.001
1	2774	2600 (93.8)	173 (6.2)	
> 1	373	330 (88.5)	43 (11.5)	
Media exposure				
No	7111	6778 (95.3)	333 (4.7)	0.903
Yes	8143	7766 (95.4)	377 (4.6)	

### 3.2 Prevalence of Self‐Reported STIs Among Women of Reproductive Age Women

**Figure 1:**
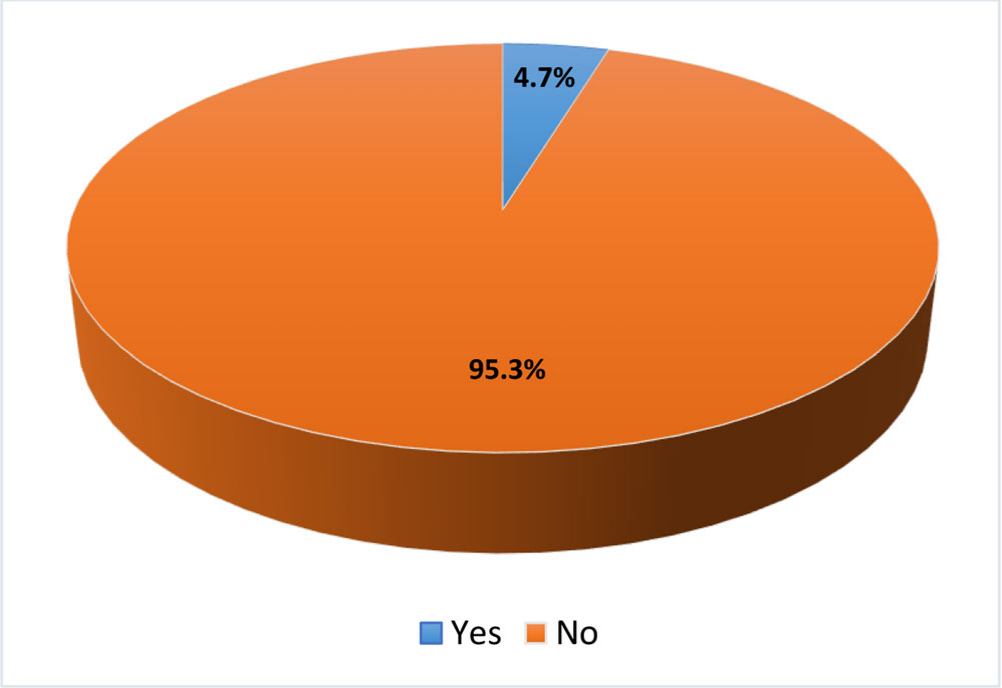
Prevalence self‐reported STIs among women of reproductive age women.

Among 15,254 women of reproductive age in Tanzania (4.7%) self‐reported having an STI in last 12 months (Figure 1).

### 3.3 Percentage of Self‐Reported STIs Among Women of Reproductive Age Women Across Different Zones in Tanzania

**Figure 2:**
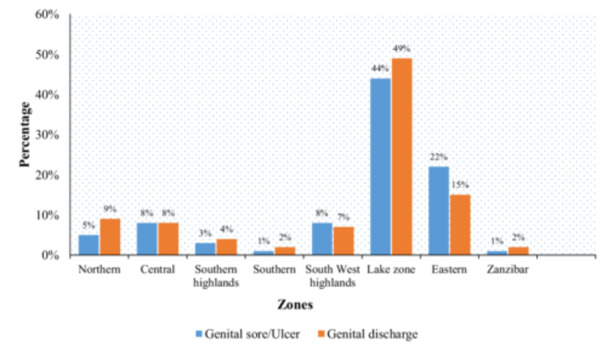
Self‐reported STIs among women of reproductive age women across different zones.

Figure 2 shows self‐reported STIs among WRA by different zones. Around half (49.2%, 43.7%) of study participants in lake zone had STIs genital sores and genital discharge, respectively. And the southern zone consists of a low percentage of STIs with only (0.9%, 1.7%) genital sores and genital discharge respectively, followed by Zanzibar with (1.4%, 1.9%) of genital sores and genital discharge respectively.

### 3.4 Factors Associated With Sexual Transmitted Infections Among Reproductive Age Women

Maternal age, wealth status, marital status and number of sexual partners were associated with sexual transmitted infections in the adjusted modified poison regression at a *p* value of < 0.05. Women aged 25–34 years of age had higher likelihood of STIs compared to younger ones (ARR = 1.55, CI: 1.22–1.95). Compared to women from poor households, women from rich households were associated with higher likelihood of acquiring STIs (ARR = 1.37, CI:1.05–1.79). Participants who were married had 19% less likelihood of sexual transmitted infections (ARR = 0.81, 95% CI: 0.65–0.99) compared to unmarried. Women with 2+ number of sexual partners had higher likelihood of STIs (ARR = 1.80, 95% CI: 1.24–2.63), compared to those who had none (Table 2).

**Table 2:** Factors associated with sexual transmitted infections among reproductive age women in Tanzania (*n* = 15,254).

Variable	Crude RR (95% CI)	Adjusted RR (95% CI)
Age categories		
15–24	Ref	Ref
25–34	2.31 (1.84–2.89)*	1.55 (1.22–1.95)*
35–49	2.04 (1.63–2.54)*	1.39 (1.08–1.79)*
Residence		
Urban	Ref	Ref
Rural	0.82 (0.65–1.03)	0.95 (0.72–1.24)
Highest educational level		
No education	Ref	Ref
Primary	1.19 (0.94–1.51)	1.10 (0.86–1.41)
Secondary	0.98 (0.75–1.30)	1.18 (0.85–1.64)
Higher	0.98 (0.45–2.13)	0.78 (0.34–1.73)
Wealth index		
Poor	Ref	Ref
Middle	1.39 (1.08–1.79)*	1.37 (1.05–1.79)*
Rich	1.40 (1.11–1.77)*	1.39 (1.02–1.89)*
Marital status		
Unmarried	Ref	Ref
Married	1.09 (0.93–1.29)	0.81 (0.65–0.99)*
Employment status		
Not working	Ref	Ref
Working	1.40 (1.14–1.72)*	1.00 (0.82–1.24)
Condom used for the last sex		
No	Ref	Ref
Yes	1.36 (0.98–1.89)	1.14 (0.81–1.60)
Number of sexual partners		
No	Ref	Ref
1	1.53 (1.27–1.84)*	1.03 (0.81–1.32)
> 1	2.83 (1.97–4.05)*	1.80 (1.24–2.63)*
Media exposure		
No	Ref	Ref
Yes	0.99 (0.82–1.20)	0.88 (0.72–1.09)

Abbreviations: Cl, Confidence interval; RR, risk ratio.

* Statistically significant with *p* value < 0.05.

## 4 Discussion

The study aimed to determine prevalence and factors associated with STIs among WRA in Tanzania. Prevalence was found to be 4.7%. Factors that significantly associated with sexually transmitted infections among women of reproductive age in Tanzania are maternal age, wealth status, marital status and number of sexual partners.

In this study the prevalence of STIs was found to be 4.7% among WRA in Tanzania. This may reflect underreporting due to stigma, lack of awareness, limited access to healthcare and diagnostic services, additionally most of STIs are asymptomatic, so self‐reporting might underestimate the burden [2, 5, 17, 25]. Significant efforts are still needed in Tanzania to achieve the global health strategy for STIs, which envisions universal access to affordable and accessible STI prevention and treatment services [26]. Similar findings were reported by Seidu et al., and Bogale et al., where the prevalence of STIs were reported to be 4.4% and 4.3%, respectively [27, 28]. Nevertheless, this finding is inconsistent with other studies done in different parts of Sub‐Saharan countries such Kingdom of Eswatini (Swaziland) [29] and Uganda [30] which revealed high STIs prevalence of 19.4% and 26% respectively. These disparities can be explained by geographical (climatic) variations, level of poverty and marginalization different health care policies and structures and sexual networks varies between these countries [28].

Women aged 25–34 years are more likely to acquires STIs than younger ones due to a combination of behavioral, biological and social economic factors, this age group is often more sexually active, may have higher sexual activities frequency and may engage in riskier sexual behaviors such as misuse of alcohol, unprotected sex due to the need of children, and multiple sexual partners [31]. Biological susceptibility such as thinner vaginal lining and vaginal microbiota [32]. Additionally, cultural norms, urbanization and economic dependence may further contribute to the vulnerability to STIs among this age group [33]. This finding is in line with another studies done in Uganda [30], Bangladesh [34], South Africa [35] which reported similar findings, similarly, Abbai et al. reported that as a women age the likelihood of STIs decreases [36].

Furthermore, this study found that women from rich households were more likely to contract STIs compared to women from other wealth statuses, this may be linked to increased mobility, greater sexual freedom or engagement in multiple sexual partners and social media access [31, 37]. Wealth may correlate with reduced perceived risk and lower use of protective measures like condoms [38]. In some cases, affluent lifestyles can expose women to high‐risk networks where power dynamics limit safe sex practices [39]. Economic status seems to have two way effect on STI risk [37], The upstream effect of poverty can contribute to age‐disparate or transactional sexual relationships, with vulnerability heightened by restricted access to healthcare. Conversely, the downstream effect of STIs on household productivity and healthcare costs further deepens poverty [37]. Since STIs self‐reported, women from rich household are more prone to screening and more informed of the STIs due to their ability to own mass media [40]. Similarly Dadzie et al., reported that women from wealthier households were more likely to contact and report STIs [40].

Expectedly, women with two or more sexual partners were more likely to have and report STIs, having more than sexual partners increases exposure to infected individuals and the risk of transmission, it can be challenging to know and assess each partner's full sexual history and STIs can quickly spread between individuals without direct sexual contacts [41]. Similar findings were reported by other researcher in South Africa [42] Ethiopia [12] Ghana [31] and Nepal [43].

Existing evidence suggest that marriage protects acquiring STIs [3, 29, 44], the current study confirms this. It was found that married women were less likely to report STIs compared to unmarried women, this is attributed to the fact that marriage involves mutual commitment that may lower risky behaviors but also in African context married woman is expected to live cultural and societal anticipation, they are expected to modest, obedient and loyal [40]. Hence, it's not anticipated that married women would take part in multiple sexual partnerships. Another possible explanation could be the culture of STIs testing before marriage [45], that is most of the married women know their status something which decreases the likelihood of reporting STIs among married women. However, differing from our findings, studies conducted in South Africa [46] and Nepal [43] revealed that married women were more riskier and contacted and reported more STIs than unmarried women. These studies revealed that being married or cohabiting for less than 5 years significantly increases the likelihood of engaging in risky sexual behavior, aligning with patterns of sexual violence within marriage. Additionally, many women in early marriages, often arranged or imposed, may lack awareness of sexual and marital matters, making them more vulnerable to sexual abuse. Furthermore, marital quality tends to decline after the first year, and if incompatibility arises, women may lose interest in sex, potentially leading to sexual violence and behaviors that heighten their risk of STIs [42].

### 4.1 Strength and Limitation

We utilized data from the latest DHS, renowned for its robust methodologies and validation across numerous studies. The use of nationally representative data ensures that our findings are broadly applicable to Tanzanian WRA and women from other African countries. However, this study is not without limitations, relying on secondary data restricted our analysis to the variables available in the datasets, excluding crucial factors like cultural influences and patriarchal norms that may shape STI risks among women. Additionally, the cross‐sectional design of the DHS precludes causal interpretations. Self‐reported outcomes introduce the possibility of recall bias, and we urge readers to interpret the results with this in mind. While the large sample size increases the statistical power, it may also render small associations statistically significant. Despite these limitations, our findings provide valuable insights and form a foundation for further research and targeted interventions.

## 5 Conclusion and Recommendation

Despite the reported relatively low prevalence (4.7%), Tanzanian WRA are still at high risk of acquiring STIs. This study has identified specific risk factors in our community of women and they should be considered for targeted interventions among WRA. The findings demonstrate the need for commitment to further reduce the STIs prevalence and work on specific risk factors so as to attain the universal health strategy for STIs prevention and treatment which aim to significantly reduce new STI cases and related deaths. Behavioral change campaigns, young adult‐friendly health services, regular health education and community sensitization campaigns should be paired with routine STI screening programs to ensure timely identification and effective management. Strengthening rapid point‐of‐care services, including swift diagnosis using rapid tests and the provision of tailored preventive health education, is crucial. Additionally, prevention initiatives must address the unique vulnerabilities of women, including those in wealthier households, and incorporate targeted education that promotes sexual health across all relationship dynamics, regardless of partner stability. Together, these strategies can foster healthier communities and improve overall well‐being of WRA.

## Author Contributions

**Fabiola V. Moshi:** conceptualization, writing – original draft, writing – review and editing, supervision, visualization, data curation. **Jovin R. Tibenderana:** writing – original draft, methodology, writing – review and editing, formal analysis, data curation. **Thadei Liganga:** writing – original draft, writing – review and editing, visualization. **Jomo Gimonge:** writing – original draft, visualization, writing – review and editing. **Sanun Ally Kessy:** writing – original draft, writing – review and editing, methodology, formal analysis, data curation.

## Conflicts of Interest

The authors declare no conflicts of interest.

## Transparency Statement

The lead author Jovin R. Tibenderana affirms that this manuscript is an honest, accurate, and transparent account of the study being reported; that no important aspects of the study have been omitted; and that any discrepancies from the study as planned (and, if relevant, registered) have been explained.

## Data Availability

The data that support the findings of this study are openly available in Demographic Health Survey at https://dhsprogram.com/.
